# Spider–Plant Interaction: The Role of Extrafloral Nectaries in Spider Attraction and Their Influence on Plant Herbivory and Reproduction

**DOI:** 10.3390/plants13030368

**Published:** 2024-01-26

**Authors:** Karoline Pádua de Oliveira Dias, Vanessa Stefani

**Affiliations:** 1 Postgraduation Program in Ecology, Conservation and Biodiversity, Federal University of Uberlândia, Uberlândia 38405-302, Brazil; karol.padua15@gmail.com; 2 Laboratory of Venomous Arthropods of the Cerrado, Institute of Biology, Federal University of Uberlândia, Uberlândia 38405-302, Brazil; 3 Laboratory of Natural History and Reproduction of Arthropods (Laboratório de História Natural e Reprodutiva de Artrópodes/LHINRA), Institute of Biology, Federal University of Uberlândia, Uberlândia 38405-302, Brazil

**Keywords:** *Heteropoterys pteropetala*, biotic defence, Cerrado, plant fitness, facultative mutualism, neutral effect

## Abstract

Spiders, abundant and diverse arthropods which occur in vegetation, have received little attention in studies investigating spider–plant interactions, especially in plants which have extrafloral nectaries (EFNs). This study examines whether spiders attracted to EFNs on the plant *Heteropterys pteropetala* (Malpighiaceae) function as biological protectors, mitigating leaf herbivory and positively impacting plant fitness, through manipulative experiments. Spiders are attracted to EFNs because, in addition to consuming the resource offered by these structures, they also consume the herbivores that are attracted by the nectar. At the same time, we documented the reproductive phenology of the plant studied and the abundance of spiders over time. Our results revealed that the plant’s reproductive period begins in December with the emergence of flower buds and ends in April with the production of samarids, fruits which are morphologically adapted for wind dispersal, aligning with the peak abundance of spiders. Furthermore, our results demonstrated that spiders are attracted to plants that exude EFNs, resulting in a positive impact on reducing leaf area loss but with a neutral effect on protecting reproductive structures. By revealing the protective function of spiders’ vegetative structures on plants, this research highlights the ecological importance of elucidating the dynamics between spiders and plants, contributing to a deeper understanding of ecosystems.

## 1. Introduction

Interactions between spiders and plants can provide evidence of the existence of facultative mutualistic relationships, influencing the structure of ecological communities and the fitness of plants [1,2,3,4]. Understanding the function of each organism in this interaction will allow for a better understanding of the evolutionary paths that lead to mutualism. Spiders are considered excellent predators, and, when they live on plants, they forage for their prey. In addition, spiders occasionally use plant resources to supplement their insect-based diet [5,6,7,8]. Among these plant-supplied foods are extrafloral nectaries (EFNs) [8,9]. Thus, spiders provide various services to plants, including protection against leaf and flower herbivores, consequently reducing leaf herbivory and/or increasing seed production [1,10,11,12], acting as important biological defenders [13]. Although spiders are traditionally considered predators in ecosystems, these organisms also frequently feed on the products of extrafloral nectaries [14]. Biotic defences have a mutualistic character whereby resources such as plant EFNs are exchanged for spider services and are mediated by the interests, costs, and benefits for both groups [8]. The costs and benefits of this association can vary depending on various factors, such as the identity of the spider family [15], the season of EFN activity, and even the presence of competitors such as ants [1,2,16]. However, the relationship between spiders and plants can also be negative, as spiders consume or interfere with pollinators, leading to a reduction in plant fitness [17,18].

EFNs are nectar-producing structures not associated with pollination and found in various parts of plants, such as leaves, stems, and flower bud calyxes [19,20]. These nectaries produce a solution rich in water, sugars, amino acids, proteins, and lipids [21,22]. EFN-bearing plants are common in the Cerrado biome [23,24]. A study by Nahas et al. [14] found the presence of fructose from EFNs from eight different plant species in 39 spider species from seven families. This indicates that feeding on EFNs is advantageous for spiders, as nectar is an excellent source of energy [1,25,26,27].

Although spiders are among the most abundant and diverse arthropods in vegetation, studies on their interactions with plants are relatively scarce, and the literature on integrative studies on the relationship between spiders and extrafloral nectaries is still limited [28,29]. In this context, the aim of this study is to determine whether or not the presence of extrafloral nectaries on *Heteropterys pteropetala* A. Juss. (Malpighiaceae) is an attractive factor for spiders and whether these animals act as biological protectors. Our main hypothesis is that spiders are attracted to the extrafloral nectaries on this plant and, consequently, that their presence reduces leaf damage and increases the reproductive success of *H. pteropetala* (Table 1). Spiders can reduce damage to leaves and increase the reproductive success of a plant by consuming the herbivores present on it. In addition, we describe the phenology of the different reproductive phases of *H. pteropetala* and the abundance of spiders found over time.

## 2. Results

**Figure 1:**
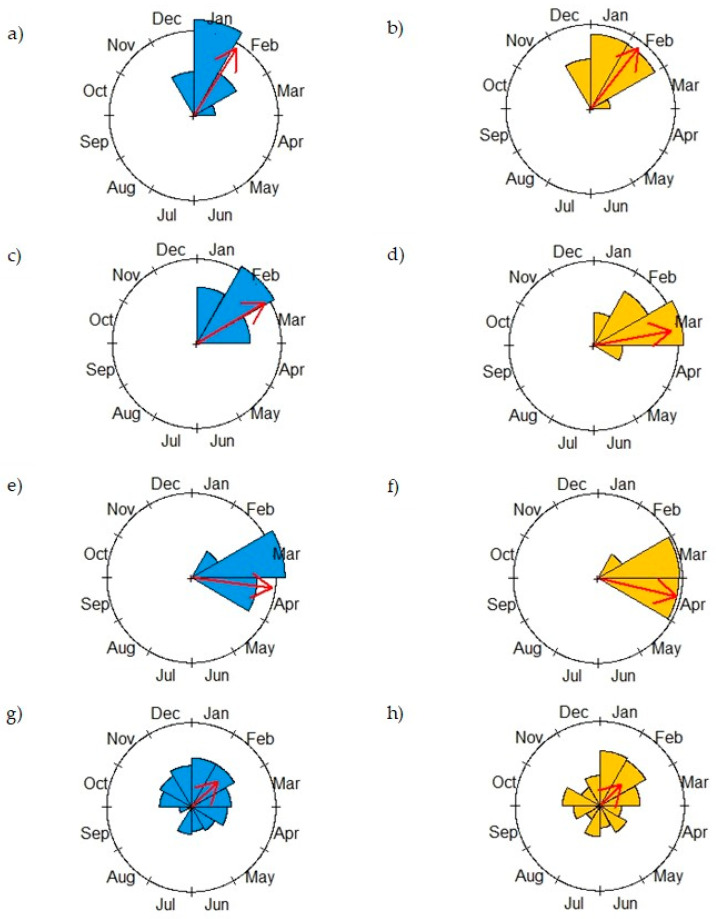
Number of reproductive structures produced by *Heteropoterys pteropetala* and spider abundance between December 2021 and January 2022 for the EFNs_active_ (blue, left) and EFNs_inactive_ (yellow, right) groups: (**a**,**b**) floral buds; (**c**,**d**) inflorescences; (**e**,**f**) samarids; and (**g**,**h**) spider abundance. Arrow position represents the mean vector (µ), and arrow length represents the length of the mean vector (r).

The reproductive period of the H. pteropetala in our study began in December 2021 with the emergence of the first floral buds and ended in May 2022 with the collection of the samarids. The reproductive peak for the floral buds in both groups (EFNs_active_ and EFNs_inactive_) was in February (Rayleigh test for buds with EFNs_active_ z = 0.94; *p* < 0.001; buds with EFNs_inactive_ z = 0.91; *p* < 0.001) (Table 2, Figure 1a,b). The inflorescences on the plants EFNs_active_ reached their peak in February (Rayleigh test: z = 0.943; *p* < 0.01), while the EFNs_inactive_ plants reached their inflorescence peak in March (Rayleigh test: z = 0.932; *p* < 0.01) (Table 1, Figure 1c,d). The peak of samarid production occurred in April for both manipulations (Rayleigh test for samarids with EFNs_active_ z = 0.957; *p* < 0.01; samarids with EFNs_inactive_ z = 0.952; *p* < 0.01) (Table 1, Figure 1e,f). Spider abundance was higher in January and February, with peak abundance being observed in February in both manipulations (EFNs_active_ z = 0.436; *p* < 0.01; and EFNs_inactive_ z = 0.366; *p* < 0.03) (Table 1, Figure 1g,h). There was an absence of spiders on the EFNs_active_ plants in August, and the lowest abundance of spiders was also recorded in August for the EFNs_inactive_ plants.

**Table 2:** Circular statistics applied to reproductive phenophases and spider abundance in *Heteropoterys pteropetala* with EFNs_active_ (*n* = 20) and EFNs_inactive_ (*n* = 20) in a Cerrado area at the Ecological Reserve of Clube Caça e Pesca Itororó in Uberlândia, Minas Gerais, Brazil. The Rayleigh test was conducted with a significance level of 0.05.

	Reproductive Phenophases						Abundance	
	Floral Buttons EFNs_active_	Floral Buttons EFNs_inactive_	Flowers EFNs_active_	Flowers EFNs_inactive_	Samarids EFNs_active_	Samarids EFNs_inactive_	Spiders EFNs_active_	Spiders EFNs_inactive_
Abundance total	15,368	14,734	7183	13,140	24,105	24,620	97	60
Length of mean vector (r)	0.49	0.44	0.51	0.85	0.77	0.84	0.43	0.37
Mean vector (µ)	32.15	34.1	59.4	71.3	97.8	102.2	42.7	41.65
Month	February	February	February	March	April	April	February	February
Rayleigh test (Z)	0.94	0.91	0.943	0.932	0.957	0.952	0.436	0.366
Rayleigh test *(p*)	0.001	0.001	0.001	0.001	0.001	0.001	0.01	0.03

**Figure 2:**
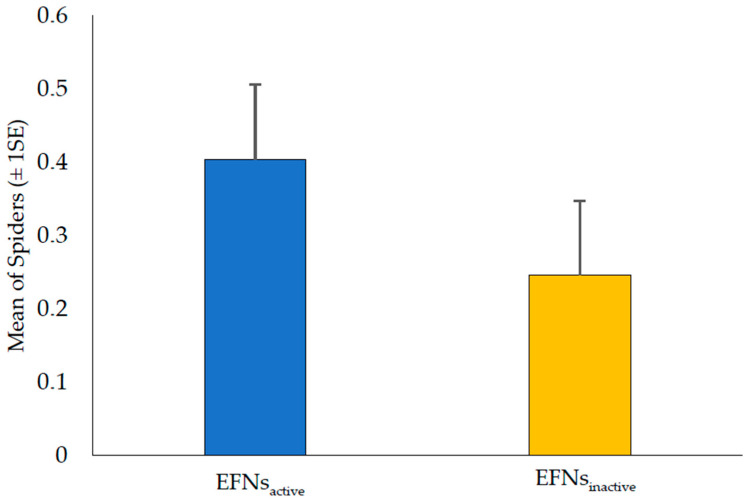
Mean number of spiders (±1 SE/standard error) per plant of *Heteropoterys pteropetala* with and without active EFNs (Extrafloral Nectaries).

Spider abundance was higher in the EFNs_active_ plants than in the EFNs_inactive_ plants (χ^2^ = 9.0681; df = 1; *p* = 0.0026, Figure 2). A total of 157 spider specimens were counted, with 97 individuals found on the EFNs_active_ plants and 60 found on the EFNs_inactive_ plants (Table 3). The EFNs_active_ plants had a higher number of spiders from the families Thomisidae, Araneidae, and Salticidae compared to the EFNs_inactive_ plants (Table 3). However, only the Cheiracanthiidae family had a higher number of representatives in the EFNs_inactive_ plants compared to the EFNs_active_ plants (Table 3). The families Theridiidae and Oxyopidae showed no significant difference in the number of individuals between the treatment and control plants. A total of 470 insects were seen to be associated with *H. pteropetala* in both treatments, with the orders Lepidoptera (larval stage) and Hemiptera being the most abundant, especially in plants with EFNs_inactive_ (Table A1).

**Table 3:** Abundance of spider families found in different manipulations of the plant *Heteropoterys pteropetala*.

Family	Number of Individuals on Plants with EFNs_active_	Number of Individuals on Plants with EFNs_inactive_	X^2^	*p*
Thomisidae	47	27	7.33	0.026
Araneidae	22	7	9.88	0.014
Salticidae	13	7	8.87	0.042
Theridiidae	9	6	2.21	0.652
Cheiracanthiidae	4	12	8.21	0.041
Oxyopidae	2	1	0.24	0.423
Total	97	60		

**Figure 3:**
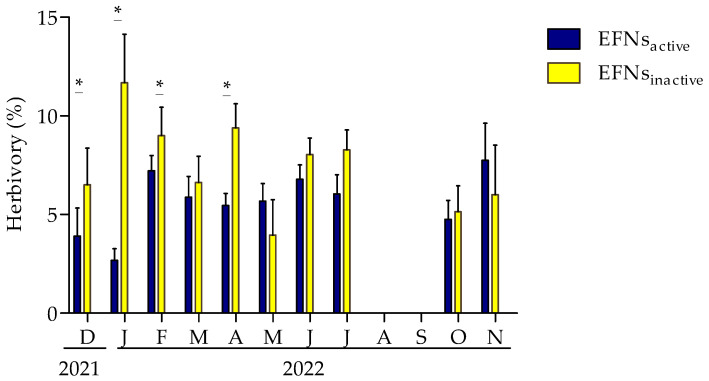
Foliar area loss (mean ± SE/standard error) in *Heteropoterys pteropetala* from December 2021 to November 2022. The response variable was herbivory; plant type (EFNs_active_ and EFNs_inactive_) was the predictor variable; months of the year and plant identification were the random variables. * = significant difference between the manipulations (Tukey’s post hoc: *p* < 0.05).

The EFNs_inactive_ plants showed less loss of leaf area (χ^2^ = 35.646; df = 1; *p* = 0.0019) and less variation in herbivory within the manipulated groups (χ^2^ = 11.609; df = 1; *p* = 0.0065), and this variation persisted over the months studied (χ^2^ = 13.206; df = 1; *p* = 0.0001) compared to the EFNs_inactive_ plants. There was also variation in the herbivory between different plant manipulations in December (2021), January, February, and April (2022) (Figure 3). In August and September, the plants were leafless, which resulted in a lack of herbivory, and they only began to sprout again in September (Figure 3). The samarid/bud ratios, samarid/flower ratios, and seed weight did not differ significantly between the EFNs_active_ and EFNs_inactive_ plants (Table 4).

**Table 4:** Productivity of *Heteropoterys pteropetala* in the presence and absence of spiders indicated by the ratio of samarids/buds, samarids/flowers, and seed weight. Values represent mean ± SE (standard error).

	EFNs_active_	EFNs_inactive_	X^2^	*p*
Samarids/Buds (Gamma)	1.7 ± 0.13	2 ± 0.13	0.957	0.328
Samarids/Flowers (Gamma)	0.384 ± 0.132	0.563 ± 0.132	2.36	0.12
Seed Weight (Gaussian)	23.08 ± 2.28	26.16 ± 2.28	0.0672	0.795

## 3. Discussion

Our study suggests that spiders are attracted to plants that exude EFNs and that this attraction has a positive effect on reducing leaf area loss for *H. pteropetala* plants in the Brazilian Cerrado, confirming the first and second hypotheses. Thus, we have demonstrated that the presence of active nectaries acts as a source of attraction for spiders that can act as efficient biological protectors. In other words, the nectar produced in EFNs can complement the diet of arthropod predators [26,27] and consequently attract these organisms, leading to a reduction in herbivory [30].

In particular, we found that active EFNs more efficiently attract spiders belonging to the Thomisidae, Araneidae, and Salticidae families. Similar results were obtained by Stefani et al. [31], who, after isolating shrubs of *Palicourea rigida* Kunth (Rubiaceae) from ants and measuring the recruitment of spiders visiting post-floral nectaries, found representatives of the Thomisidae and Salticidae families to be among those most abundant. This abundance of spiders may be associated with both the attractiveness of the nectar and the absence of ants. Supposedly, the great challenge faced by different species of spiders that use EFNs as a source of complementary food is to break through the defenses promoted by ants [2,16]. The greatest abundance of spiders in our study was recorded during the reproductive period of *H. pteropetala* in both manipulations (Figure 1), although the greatest abundance was observed in the EFNs_active_ plants (Figure 1 and Figure 2; Table 2). This increase in abundance (in both manipulations) may have influenced neutral protection rates during this period, refuting our third hypothesis. The presence of reproductive structures, such as buds, flowers, and fruits (forming samarids), can provide spiders with a wide variety of shelters, opportunities to find conspecifics, anchoring points for webs, and opportunities to use different foraging methods, even on plants with inactive EFNs [11,32].

Of all the spider families found, the Thomisidae family was the most abundant (Table 3), found on the vegetative and reproductive parts of *H. pteropetala*. Thomisidae spiders are known to be flower spiders, as they often camouflage themselves in flower petals or structures, waiting for pollinating prey to arrive [33]. Thus, spiders from the Thomisidae family are strongly associated with the reproductive period of their host plants. Studies have shown that, when individuals from this family are present in the reproductive parts of a plant, they can have positive, neutral, or negative effects on its reproduction. For example, Romero and Vasconcellos-Neto (2003) demonstrated positive effects on the reproduction of *Trichogoniopsis adenantha* (DC) (Asteraceae) in the presence of Thomisidae spiders, as the plants with the presence of these spiders produced more seeds compared to the plants without them [34]. Neutral effects, for example, were presented by Gavini et al. (2019), who studied interactions between the flowers of *Anemone multifida* (Ranunculaceae), their floral visitors, and *Misumenops pallidus* (Thomisidae). The authors observed that the presence of spiders did not reduce the number of floral visitors or the quantity and quality of the fruit and seeds formed [12]. Finally, there is ample evidence of the negative effects that spiders have on the reproduction of their host plants. For example, in another study, the presence of Thomisidae spiders in *Leucanthemum vulgare* (Vaill.) Lam. (Asteraceae) flowers reduced the number of floral visitors and the time pollinators spent in the flowers, generating a cascade effect which resulted in a 17% reduction in fruit and seed formation [35].

Spiders from the Salticidae and Araneidae families were also more abundant on the EFNs*_active_* plants in our study. According to Jackson et al. (2001), Salticidae spiders may have the habit of feeding on nectar, indicating that nectar feeding is possibly a common behaviour in this family [27]. Orb-weaving spiders, such as Araneidae (Table 3), may also have the habit of feeding on nectar from EFNs (as well as dismantling and rebuilding their webs at regular intervals, allowing them to build their webs where resources are most abundant) [36]. For example, Nahas et al. [14] investigated the presence of fructose in the bodies of spiders that visit plants with EFNs in a neotropical savannah environment. In their study, the species *Araneus venatrix* (Araneidae), collected at night from *Qualea grandiflora* (Vochysiaceae) plants, showed the highest concentrations of fructose [14]. Thus, araneids build their webs to capture their prey on plants with EFNs and supplement their diet with nectar. The arrival of new herbivores on the plant can occur by air, meaning that the webs built on the plant capture these herbivores before they even reach the plant, reducing the damage caused by herbivory.

Unlike the other spider families found on *H. pteropetala*, the Cheiracanthiidae family was more abundant on the EFNs*_inactive_* plants than on the EFNs*_active_* plants, despite the fact that this family is known for its nectar consumption [36]. As the representatives of the Cheiracanthiidae family were adults and in the oviposition period, they were possibly found in greater numbers on the EFNs*_inactive_* plants because these locations allowed them to avoid the presence of competing spiders and probable predators of their eggs and young. In addition, spiders during egg sac care reduce their consumption of prey [37], thus affecting any positive interaction with the plant.

In summary, spiders are attracted to EFN nectar, confirming the existence of mutualism in the form biotic protection between spiders and *H. pteropetala*. However, the positive relationship is limited to leaf structures, while, in the reproductive parts, the association found was neutral. Thus, the predatory activity of spiders on reproductive structures suggests a commensal role in which one species (spider) benefits from the interaction, but the other (plant) is neither benefited nor harmed.

## 4. Materials and Methods

### 4.1. The Study Site and Species of Plant

This study was conducted from December 2021 to November 2022 at the Ecological Reserve of the Clube de Caça e Pesca Itororó de Uberlândia (18°59′ S and 48°18′ W, WGS84 Datum, ~640 ha), in the state of Minas Gerais (MG), Brazil. The reserve’s vegetation comprises various savanna physiognomies, with trees reaching up to 8 m in height [38]. The mean monthly rainfall ranges between 0 and 360 mm, and the mean monthly temperature is between 20.0 and 25.5 °C, with a dry season between May and September and a rainy season between October and April [3,39].

**Figure 4:**
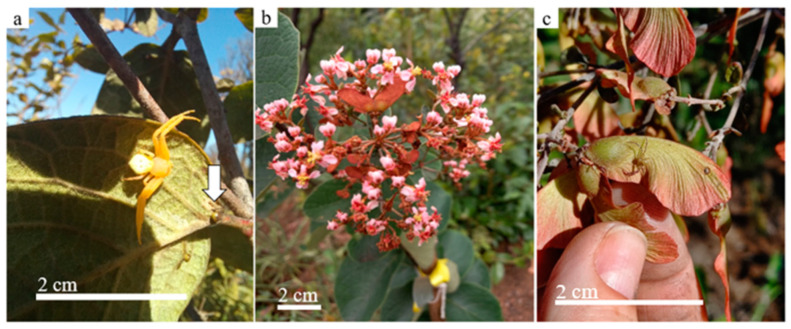
The studied plant, Heteropterys pteropetala, in the Cerrado sensu stricto at the Ecological Reserve of Clube Caça e Pesca Itororó in Uberlândia, Minas Gerais, Brazil. (**a**) Thomisidae spider on the abaxial surface of the leaf—note the white arrow indicating the pair of EFNs at the base of the abaxial region of the leaf. (**b**) Inflorescences of a studied plant. (**c**) Samarids with the presence of a juvenile Thomisidae spider.

The plant species studied, *Heteropterys pteropetala*, is a shrub approximately 2 m tall, with two extrafloral nectaries (EFNs) at the base of each leaf (Figure 4a), at the base of the pedicel of the flower buds, and on the bracts of the inflorescences [40]. The inflorescences are terminal panicles with pink flowers (Figure 4b) and are zygomorphic, with five petals and five sepals, and, at the base of each sepal, there are two elaiophores (oil glands), totalling between eight and ten glands per flower [41]. Each flower can produce up to three samarids (a fruit morphologically adapted for wind dispersal) (Figure 4c) [42]. *H. pteropetala* is dependent on cross-pollination for fruiting and is an important species for studies of ecological interactions due to the diversity in its guild of floral visitors. The presence of organisms that take part in pollen transport increases the fruiting and reproductive success of the species [42].

### 4.2. Experimental Design

To test hypotheses I, II, and III (see Table 1), we isolated the plant against the presence of ants. It is known that ants are also attracted to EFNs, making them important competitors for spiders. According to a study carried out by Lange et al. [16] with nine different plant species with EFNs in a neotropical savannah area, a negative spatial/temporal effect of spider abundance was observed in the presence of ants. In addition, Stefani et al. [2] observed that spider species’ richness was significantly higher in the absence of ants, although the reverse was not true, possibly due to the different species composition of the ants and spiders found and, consequently, the different types of interactions between them. Thus, the absence of ants in this study was necessary so that these organisms would not influence our results. Non-toxic resin (entomological glue—Tanglefoot^®^) was added to the base of the trunk of all the plants to prevent ants from accessing the plant. All the structures, such as grasses, that could serve as a bridge for the ants to access the plants were removed. We then carried out two different manipulations on the *H. pteropetala* plants in a natural environment: (I) EFNs*_active_* plants were individuals with active extrafloral nectaries (*n*= 20); and (II) EFNs*_inactive_* plants were individuals with inactive extrafloral nectaries (*n* = 20). The plants in the EFNs*_inactive_* group underwent a process of enamelling all the nectaries, blocking them, and preventing the release of nectar—in other words, making them inactive. In the plants in the EFNs*_active_* group, glaze was also applied to the abaxial part of the leaf, next to the extrafloral nectary, allowing the normal release of nectar. Weekly inspections were carried out on all the plants to check the integrity of the nectary obstructions in the EFNs*_inactive_* plants, as well as the entomological resin at the base of the trunk in both manipulations, to prevent ant access. To describe the reproductive phenology of *H. pteropetala*, all flower buds, inflorescences, and samarids were quantified weekly during the plants’ reproductive period.

To test hypothesis I, all the experimental plants were inspected weekly; the spiders found were photographed and quantified after a visual search of the entire bush. The branches were also shaken over a white tray, so that, if any animals were not found during the visual sweep, they would be on the tray for quantification. After the procedure, all the spiders were placed back on the plant.

To test hypothesis II, herbivory rates were measured monthly on five leaves of each plant in both manipulations. Initially, the five leaves of each plant were marked at the initial stage of expansion to monitor and record the loss of leaf area throughout the leaves’ ontogeny, i.e., from budding to senescence. Herbivory was calculated from digital images analysed using the ImageJ software version 1.53, as performed by Calixto et al. [43].

To test hypothesis III, flower buds, flowers, and samarids were quantified weekly during the reproductive period for both manipulations. Around 30 days after flowering, the samarids were harvested, dried in the sun for a fortnight, and then weighed separately, depending on the plant manipulation, using a precision electronic analytical balance.

### 4.3. Data Analysis

All statistical analyses were performed in R version 4.2.2 (R CoreTeam, 2022). Below, we describe the packages used in each analysis.

#### 4.3.1. Phenology of *Heteropoterys pteropetala*

The data for calculating the phenology of the *H. pteropetala* plant were observed using circular statistical analyses. These analyses served to verify the occurrence of seasonality between different reproductive phenophases (presence of floral buds, flowers, and fruits) throughout the year, as well as to analyse spider abundance on plants with and without active EFNs. For the circular analyses, we divided the 360° range into 12 groups. Each group represents a month of the year, with each month corresponding to a 30° angle and the mean vector (μ) being indicative of the direction (month) where the data are possibly more concentrated (reproductive phenophases and spider abundance). Subsequently, to evaluate different plant phenophases and whether spider abundance showed a non-random distribution throughout the year, we used the Rayleigh test for uniformity after confirming the normality of the circular data [44]. In the Rayleigh test, *p*-values below 0.05 and a mean vector length (r) close to 1 indicated seasonality in the data, i.e., phenological activities were concentrated around a single period or mean angle [44]. The mean month for each variable was obtained by converting the angular mean of the corresponding mean months [44,45].

#### 4.3.2. Hypothesis I

We used “glmmTMB” [46] and “Dharma” [47] with a “Poisson” distribution to answer whether spiders are attracted to EFNs. We compared differences in spider abundance (response variable) between the plants with EFNs*_inactive_* and EFNs*_active_* (predictor variables), considering the months of the year and plant identification as random variables.

#### 4.3.3. Hypothesis II

To answer the hypothesis that spiders act in a mutualistic relationship as protectors, we analysed variations in herbivory (response variable) between the EFNs*_inactive_* and EFNs*_active_* plants (predictor variables), considering the months of the year and plant identification as random variables. We used GLMM (binomial distribution), with Tukey’s post hoc tests being performed between the manipulations. The GLMM was conducted with the “glmer” function from the “lme4” package [47], followed by “Dharma” [48] to fit and check the residuals. Model significance was analysed with the Wald χ^2^ test through the Anova function, using the “car” package [49].

#### 4.3.4. Hypothesis III

To verify whether the presence of spiders impacted the reproductive success of the plants, we used glmmTMB with a Gamma distribution to check whether EFNs*_inactive_* and EFNs*_active_* (predictor variables) influenced the proportion between samarids/buds and samarids/flowers (response variables). Plant ID was considered a random effect [49]. To analyse if there was variation in the total weight of seeds produced between the EFNs*_inactive_* and EFNs*_active_* plants, we used glmmTMB with a Gaussian distribution with the “identity” link. Individual plant identity was considered a random factor (Table 1).

## Figures and Tables

**Table 1:** Overview of the hypotheses (H) and predictions tested in this study. EFNs_inactive_ are the nectaries that were obstructed with enamel, while EFNs_active_ are the nectaries without manipulation (see methodology).

Overview	Prediction	Approach	Resource
H1: EFNs attract spiders.	EFNs_active_ plants exhibit a higher abundance of spiders compared to EFNs_inactive_ plants.	Evaluation of spider abundance between EFNs_active_ and EFNs_inactive_ plants.	Figure 1 and Figure 2 Table 2
H2: Spiders act as protectors against leaf damage.	EFNs_active_ plants have lower herbivory rates than EFNs_inactive_ plants.	Analysis of herbivory rates throughout the year between EFNs_active_ and EFNs_inactive_ plants.	Figure 3 Table 3
H3: Positive impact of spiders on plant reproductive success.	EFNs_active_ plants show a higher reproductive rate than EFNs_inactive_ plants.	Evaluation of the ratio between samarids/buds and samarids/flowers as well as the total seed weight and fruiting rate between EFNs_active_ and EFNs_inactive_ plants.	Table 4

## Data Availability

All data underlying our study was deposited in the Dryad Digital Repository https://doi.org/10.5061/dryad.v15dv423k.

## Appendix A

**Table A1:** Main insect orders found on *Heteropoterys pteropetala* in each of the manipulations.

Order	Number of Individuals on Plants with EFN_active_	Number of Individuals on Plants with EFNs_inactive_
Hemiptera	16	64
Diptera	35	18
Lepdoptera (larval stage)	134	167
Coleoptera	10	10
Hymenoptera	4	4
Orthoptera	3	5
**Total**	**202**	**268**
